# Interactions between *Lactobacillus rhamnosus* GG and oral micro-organisms in an in vitro biofilm model

**DOI:** 10.1186/s12866-016-0759-7

**Published:** 2016-07-12

**Authors:** Qingru Jiang, Iva Stamatova, Veera Kainulainen, Riitta Korpela, Jukka H. Meurman

**Affiliations:** Department of Oral and Maxillofacial Diseases, University of Helsinki and Helsinki University Hospital, P.O. Box 63, Haartmaninkatu 8, FI-00014 Helsinki, Finland; Faculty of Dental Medicine, Medical University of Plovdiv, 26 Vasil Aprilov, 4000 Plovdiv, Bulgaria; Department of Pharmacology, Medical Nutrition Physiology, Medicine Faculty, University of Helsinki, P.O. Box 63, Haartmaninkatu 8, FI-00014 Helsinki, Finland

**Keywords:** Probiotics, Biofilm, Oral pathogen, Oral health

## Abstract

**Background:**

Probiotics have shown favourable properties in maintaining oral health. By interacting with oral microbial communities, these species could contribute to healthier microbial equilibrium. This study aimed to investigate in vitro the ability of probiotic *Lactobacillus rhamnosus* GG (L.GG) to integrate in oral biofilm and affect its species composition. Five oral strains, *Streptococcus mutans*, *Streptococcus sanguinis*, *Aggregatibacter actinomycetemcomitans*, *Fusobacterium nucleatum* and *Candida albicans* were involved. The group setup included 6 mono-species groups, 3 dual-species groups (L.GG + *S. mutans*/*S. sanguinis*/*C. albicans*), and 4 multi-species groups (4/5 species and 4/5 species + L.GG, 4 species were all the tested strains except *S. mutans*). Cell suspensions of six strains were pooled according to the group setup. Biofilms were grown on saliva-coated hydroxyapatite (HA) discs at 37 °C in anaerobic conditions for 64.5 h. Biofilm medium was added and refreshed at 0, 16.5, and 40.5 h. The pH of spent media was measured. Viable cells of the 16.5 h and 64.5 h biofilms were counted. 64.5 h biofilms were stained and scanned with confocal laser scanning microscopy.

**Results:**

Our results showed that L.GG and *S. mutans* demonstrated stronger adhesion ability than the other strains to saliva-coated HA discs. L.GG, *C. albicans, S. mutans* and *F. nucleatum*, with poor ability to grow in mono-species biofilms demonstrated better abilities of adhesion and reproduction in dual- and/or multi-species biofilms. L.GG slightly suppressed the growth of *C. albicans* in all groups, markedly weakened the growth of *S. sanguinis* and *F. nucleatum* in 4sp + L.GG group, and slightly reduced the adhesion of *S. mutans* in L.GG+ *S. mutans* group.

**Conclusions:**

To conclude, in this in vitro model L.GG successfully integrated in all oral biofilms, and reduced the counts of *S. sanguinis* and *C. albicans* and lowered the biofilm-forming ability of *F. nucleatum*, but only slightly reduced the adhesion of *S. mutans. C. albicans* significantly promoted the growth of L.GG.

## Background

Probiotics, “live microorganisms that, when administered in adequate amounts, confer a health benefit on the host” [1], have shown favourable properties in maintaining oral health. Short- and long-term intake of probiotics could reduce the caries risk among children [2, 3], decrease gum bleeding and reduce gingivitis [4–6], reduce the pocket depth and positively affect the gain of clinical attachment [7], and reduce the counts of *Candida* in the elderly [8, 9]. Collective studies suggest that these positive effects are results of the interactions between probiotics and the micro-organisms harboured in individual’s oral cavity.

Micro-organisms inhabit the oral cavity in the form of biofilms (dental plaque), which progressively develop in 4 hours after meals in the absence of oral hygiene [10]. A fully developed biofilm contains micro-organisms, extracellular matrix and extracellular DNA [11]. Initial colonizers, such as streptococci and actinomyces bind to the salivary pellicle, which coats the enamel, subsequently grow together with secondary colonizers, and gradually develop biofilm communities [12]. In these mature biofilms communications of intra-species and interspecies occur on nutrition metabolism, space arrangement, and transfer of DNA [11, 13].

Daily oral hygiene, including tooth brushing and flossing, could remove most of the dental plaque, but the residual plaque, however, is still unavoidable. In the plaque, when certain harmful strains grow in greater numbers, they may contribute and cause oral diseases, such as tooth decay, periodontitis and candidiasis [14, 15]. Also poor daily oral hygiene may increase the chronic inflammatory burden to the body [16]. According to accumulating data from clinical trials, probiotics have shown capacity to be an alternative strategy for the prevention and treatment of bacterial/yeast diseases [17, 18]. In the past decade, researchers have investigated the antagonistic interactions between probiotics and pathogens in their planktonic form in broth media and/or in colonies on agars [19, 20]. Advanced biofilm models [21–24] have been built up to test their activities when grown on glass and saliva-coated hydroxyapatite (HA) surfaces. Despite great efforts, our understanding of the underlying mechanisms of probiotic behaviour is still inadequate, however. As mixed-species biofilms are undoubtedly the dominant form in nature and the oral cavity, there are pressing needs to discover behaviours of bacteria and yeasts in a more complex system. However, seldom studies investigated the effects of probiotics on multiple species biofilms. Pham et al. [25, 26] have studied the effects of *Lactobacillus rhamnosus* GG and *Lactobacillus salivarius* W24 on saliva-derived microcosmos. But no studies have reported effects of probiotics on defined multi-species biofilms, which allow us to follow the changes of each strain. Therefore, in this in vitro study we tested the abilities of six strains to form and build up biofilms on saliva-coated HA discs in six mono-species groups, in three dual-species groups (L.GG + *S. mutans*/*S. sanguinis*/*C. albicans*), and in four multi-species groups (4/5 species and 4/5 species + L.GG, 4 species were all the tested oral strains except *S. mutans*), respectively. We aimed to explore the ability of probiotic *Lactobacillus rhamnosus* GG to integrate in biofilms and influence its species composition in multiple species biofilms.

## Results

### Growth

**Fig. 1:**
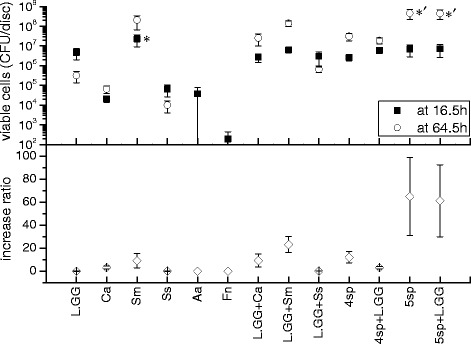
Total number of viable cells (TNVC) from biofilms and the increase ratios in self-development stage. TNVC of Sm group at 16.5 h was significantly higher (*P* < 0.05) than TNVC of the rest of the groups at 16.5 h, and marked as ‘*’. TNVC of 5sp and 5sp + L.GG at 64.5 h were significantly higher (*P* < 0.05) than TNVC of the rest of the groups (except Sm group) at 64.5 h, and marked as ‘*'’. Data represent the means ± SDs

**Fig. 2:**
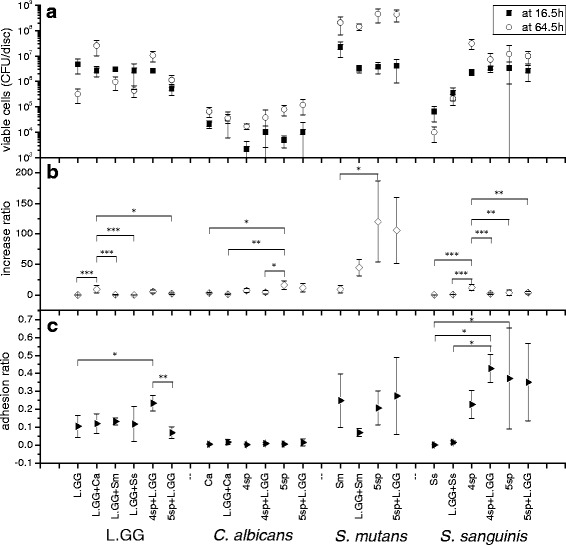
Viable cells, increase ratio, and adhesion ratio of each strain in all the groups. **a** Viable cells of each strain in each group from 16.5 h and 64.5 h biofilms; **b** viable cells increase ratio of each strain in each group in self-development stage; **c** cell adhesion ratio of each strain to saliva-coated HA discs in adhesion stage. Data represent the means ± SDs. *P < 0.05, **P < 0.01, ***P < 0.001

The growth abilities of the six strains in 13 groups are presented in Figs. 1 and 2. L.GG, *C. albicans*, *S. mutans*, and *S. sanguinis* were able to build up biofilms in mono-species culture after three days cultivation, but *A. actinomycetemcomitans* and *F. nucleatum* were not. They were detected only at 16.5 hours. The total numbers of viable cells in dual- and multi- species groups were generally higher than in mono-species groups. The greatest cell numbers from 64.5-hour-old biofilms appeared in groups 5sp and 5sp + L.GG (4.6 ± 2.4 × 10^8^ and 4.5 ± 2.3 × 10^8^ CFU/disc), and the cell numbers were significantly higher (*P* < 0.05) than in all other groups (L.GG 3.2 ± 1.8 × 10^5^, Ca 6.6 ± 2.8 × 10^4^ , Ss 1.0 ± 0.6 × 10^4^, L.GG + Ca 2.6 ± 1.6 × 10^7^, L.GG + Sm 1.4 ± 0.4 × 10^8^, L.GG + Ss 6.5 ± 2.0 × 10^5^, 4sp 3.1 ± 1.3 × 10^7^, 4sp + L.GG 1.9 ± 0.7 × 10^7^ CFU/disc), except group Sm (2.1 ± 1.4 × 10^8^ CFU/disc).

Increase ratios of total viable cells in groups of 5sp and 5sp + L.GG were 65.0 ± 33.9 and 61.3 ± 31.3, which were higher (not significantly, NS) than in the other groups. The ratio was 12.1 ± 5.0 in group 4sp, while it decreased to 3.2 ± 1.1 in the presence of L.GG. This increase ratio was lower (NS) than 1.0 in the groups L.GG (0.067 ± 0.038), Ss (0.153 ± 0.092) and L.GG + Ss (0.216 ± 0.064), respectively.

L.GG grew best in the presence of *C. albicans*, and the corresponding increase ratio for L.GG was 9.5 ± 5.8, which was significantly higher (*P* < 0.05) than increase ratio in the other groups, except in group 4sp + L.GG (5.7 ± 2.2).

**Fig. 3:**
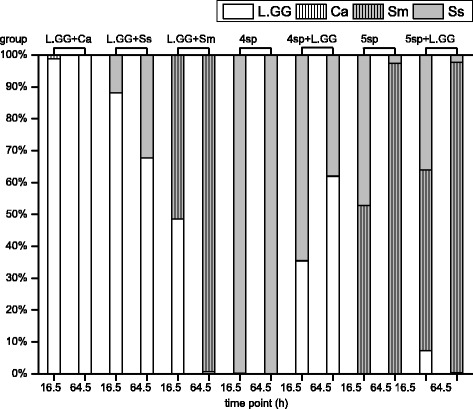
Viable cell number (%) of the cultured strains. The cultured strains were L.GG, *C. albcians* (Ca), *S. mutans* (Sm), and *S. sanguinis* (Ss), in dual- and multi-species groups at 16.5 h and 64.5 h biofilms

*C. albicans* grew similarly in all groups. The highest increase ratio of viable *C. albicans* cells was detected in group 5sp (16.1 ± 7.2), and the ratio slightly decreased to 11.7 ± 7.0 when *C. albicans* was co-cultured with L.GG. This decrease of *C. albicans* increase ratio also appeared in other pair groups, when L.GG was involved in the culture, namely groups Ca (3.20 ± 1.33) and Ca + L.GG (1.05 ± 0.41), groups 4sp (7.11 ± 3.28) and 4sp + L.GG (4.63 ± 2.83). Percentage of the cell number of *C. albcians* in L.GG + Ca at time point of 64.5 h was decreased compared with the percentage at 16.5 h (see Fig. 3).

*S. mutans* grew well in each group, and the viable cell numbers from all 64.5 h-old biofilms reached the same level of 10^8^ CFU/disc. A slight decrease in numbers of *S. mutans* viable cells from 16.5 h-old biofilm was found in L.GG + Sm biofilm (3.2 ± 1.0 × 10^6^ CFU/disc), compared with its numbers in mono culture (2.2 ± 1.3 × 10^7^ CFU/disc). The increase ratio of *S. mutans* in the 5 species biofilm was significantly higher (*P* < 0.05) than *S. mutans* alone.

*S. sanguinis* grew better in multi-species groups compared to its growth in mono-species biofilm. The best growth was seen in 4sp group, but it was supressed by L.GG. In 4sp group, the number of viable cells from 64.5 h-old biofilm was 3.1 ± 1.3 × 10^7^ CFU/disc and the increase ratio was 12.10 ± 4.99. But these values were significantly (*P* < 0.05) decreased to 0.7 ± 0.5 × 10^7^ and 1.90 ± 1.30 when L.GG was inoculated to the 4 species biofilm. Percentage of cell numbers of *S. sanguinis* in 4sp + L.GG at 64.5 h (38 %) was smaller than the percentage at 16.5 h (65 %) (Fig. 3). And numbers of viable *S. sanguinis* cell from 64.5 h-old biofilms were lower than 1.2 × 10^7^ and the increase ratios were lower than 4.0 in all the other groups.

*A. actinomycetemcomitans* and *F. nucleatum* were part of the cell suspensions inoculated to the multi-species biofilm system at 0 h, but their numbers were undetectable by the method used in this study and thus not compared with the other strains in Figs. 1, 2, and 3. No viable cells were detected from the negative control biofilms, which were cultured with physiological saline on saliva-coated HA discs.

### Adhesion ratio

The highest adhesion ratio of L.GG appeared in group 4sp + L.GG (0.234 ± 0.043) and lowest in group 5sp + L.GG (0.070 ± 0.033). The adhesion ratios of *C. albicans* were slightly higher in the presence of L.GG, namely 0.017 ± 0.014 (L.GG + Ca) and 0.005 ± 0.002 (Ca), 0.010 ± 0.007 (4sp + L.GG) and 0.003 ± 0.003 (4sp), and 0.015 ± 0.020 (5sp + L.GG) and 0.006 ± 0.003 (5sp), respectively. For *S. mutans*, this ratio, when co-cultured with L.GG (0.07 ± 0.02), was only one third of the value in mono culture biofilm (0.25 ± 0.15). In group 4sp + L.GG the adhesion ration of *S. sanguinis* was 0.428 ± 0.077 and significantly higher (*P* < 0.05) than that in mono- and dual- species biofilm, 0.001 ± 0.001 and 0.160 ± 0.008, respectively.

### pH values of spent media

**Fig. 4:**
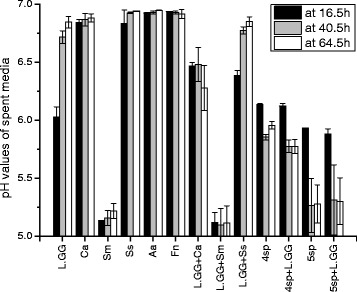
pH values of spent media in each group at 16.5 h, 40.5 h and 64.5 h. Data represent the means ± SDs

To describe the environments where biofilms grew in, pH values of each spent media was measured and shown in Fig. 4. The pH values in groups Sm and L.GG + Sm varied from 5.1 to 5.2, which were significantly lower (*P* < 0.001) than in the rest of the 11 groups, respectively at 16.5 h, 40.5 h and 64.5 h time points. The pH values of L.GG in a mono culture decreased to 6.03 ± 0.09 at 16.5 h, and returned to 6.72 ± 0.05 at 40.5 h and 6.84 ± 0.05 at 64.5 h. Groups Ca and Ss showed similar behaviour, but the pH decrease at 16.5 h was lower than 0.17. Values from groups Aa and Fn were stable in all the three time points (6.92).

Multi-groups generally showed greater reductions of pH values than mono groups, except group Sm. The pH values in the multi-species groups varied from 5.9 to 6.1 at 16.5 h. At 40.5 and 64.5 h, pH values decreased to ~5.3 in 5sp and 5sp + L.GG, which were significantly lower (*P* < 0.05) than the values in 4sp and 4sp + L.GG (5.7-5.9).

Three dual-species groups (L.GG + Ca/Sm/Ss) showed different changes of pH values. The pH values were the lowest in L.GG + Sm (5.11-5.12). In the L.GG + Ca group the value was 6.46 ± 0.03 at 16.5 h, 6.48 ± 0.15 at 40.5 h, and decreased to 6.28 ± 0.19 at 64.5 h. The pH values in L.GG + Ss were 6.39 ± 0.04 at 16.5 h, 6.77 ± 0.03 and 6.85 ± 0.04 at all the three time points.

### CLSM images

**Fig. 5:**
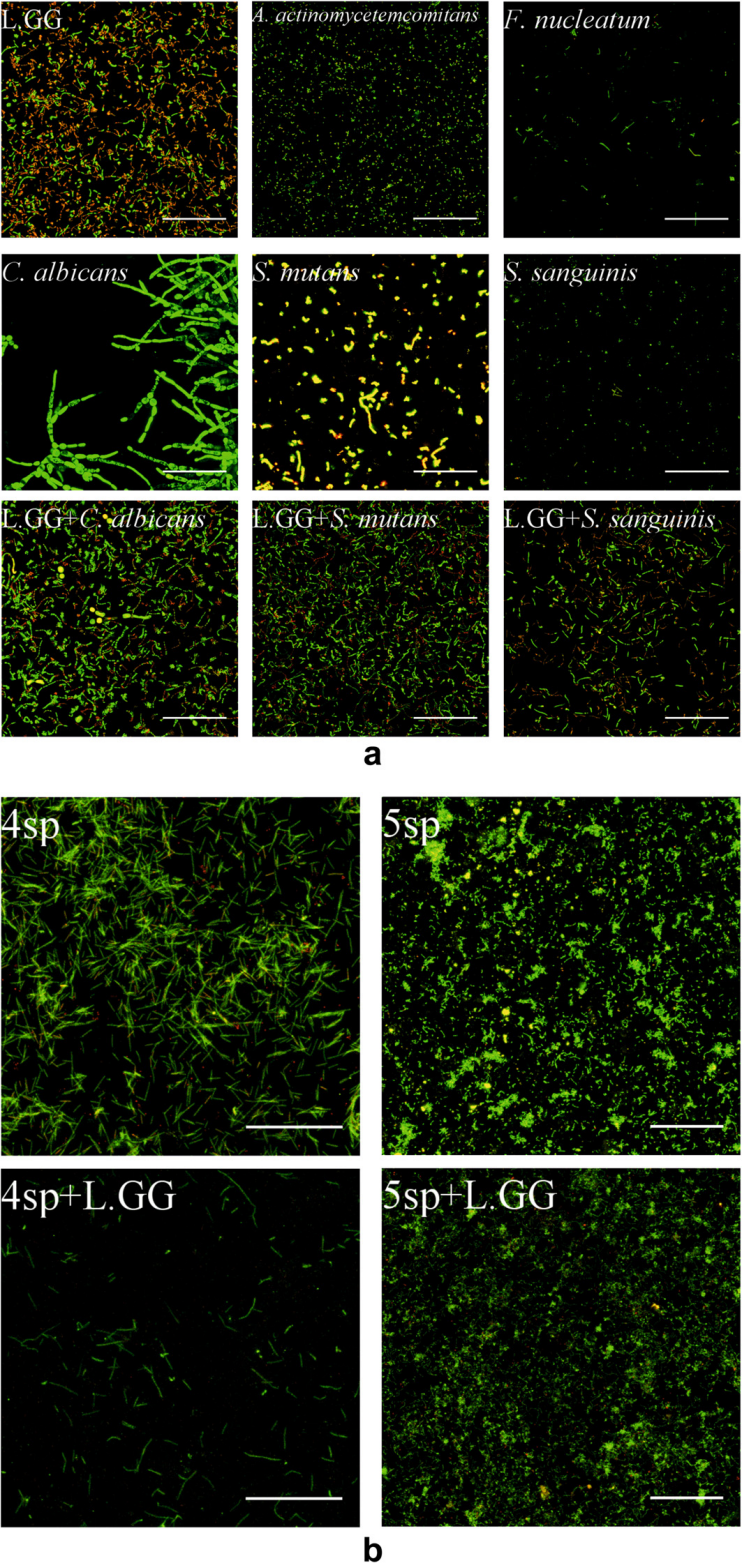
**a** Maximum intensity projection of CLSM images. CLSM images of 64.5 h bioiflms from mono- and dual-species groups stained with LIVE/DEAD® BacLight™ Bacterial Viability Kit. Live cells in green were stained with Syto 9 and dead cells in red were stained with propidium iodide. Images were obtained with a 63× glycerol immersion objective. Scale bar is 50 μm. **b** Maximum intensity projection of CLSM images. CLSM images of 64.5 h bioiflms from multi-species groups stained with LIVE/DEAD® BacLight™ Bacterial Viability Kit. Live cells in green were stained with Syto 9 and dead cells in red were stained with propidium iodide. 4sp = *C. albicans* + *A. actinomycetemcomitans* + *F. nucleatum* + *S. sanguinis*. 5sp = 4sp + *S. mutans*. Images were obtained with a 63× glycerol immersion objective. Scale bar is 50 μm

From the CLSM images shown in Fig. 5a and 5b, the cell morphology of each strain in mono-species biofilms was able to be observed. In all dual-species groups, L.GG established well, which increased the difficulty to distinguish the cells of *C. albicans*, *S. mutans*, and *S. sanguinis* in the biofilms. The biofilms of 4sp group were mostly covered by *F. nucleatum*, and some of the cells were clearly seen in clusters, but *F. nucleatum* was sparsely attached to saliva-coated HA discs in the 4sp + L.GG group. In the biofilms images of 5sp and 5sp + L.GG, the cells grew in clusters and made it difficult to see the differences between the two groups. No cells were scanned in the negative control biofilms.

## Discussion

This in vitro study aimed to test if L.GG could establish in oral biofilms and intervene with their compositions. We built 64.5 h mono-, dual-, and multi-species biofilms. Our results show that L.GG was able to retain and proliferate in in vitro oral biofilms, and showed various effects on the growth of the 5 strains investigated in the biofilm models.

The ability to attach and develop biofilms in mono-culture was highly strain dependent. L.GG and *S. mutans* demonstrated stronger ability than the other strains (namely *S. sanguinis*, *C. albicans*, *A. actinomycetemcomitans*, and *F. nucleatum*) to adhere to saliva-coated HA discs in the model. L.GG, a well-studied strain, has been shown to be able to colonise the oral cavity for at least two weeks after discontinuation of consumption of the yoghurt [27]. And it has displayed good in vitro adherence not only to epithelial cells and mucus but also to abiotic surfaces [28], which agrees with our result. But Lebeer et al. also have pointed out that in vitro biofilm formation by L.GG was strongly modulated by culture medium factors. The explanation of the reduced viable counts of L.GG from 64.5 h biofilm in our study is unclear. *S. mutans* showed the strongest adhesion ability, whereas *S. sanguinis* was sparsely bound to saliva-coated HA discs, and showed decreased counts of viable cells after the self-development stage. Streptococcal species have been reported being one of the many etiological factors of dental caries, and have been considered as pioneer colonizers settled down to salivary proteins and glycoproteins adsorbed on tooth enamel [29]. *S. mutans* has a greater ability to form biofilm than the isolates of other *Streptococcus* species colonising the human oral cavity [30], which agrees with our result.

*C. albicans* showed good potential to build up the biofilms in mono culture, as the viable cells from biofilms grew twice more during the latter 48 h cultivation, although the numbers were much lower compared to the other strains at each time point. The original inoculum of *C. albicans* was ten times lower than the other strains under the same optical density at wave length of 490 nm (OD_490_), but its cell size was two times bigger than that of the other strains. In addition, different shapes of *C. albicans* were observed from CLSM images. *C. albicans*, causing oral candidiasis, has been reported a polymorphic organism that can grow as yeast, pseudohyphae, and hyphae; and *Candida* pathogenesis can be established by virtue of *Candida* growth and yeast-to-hyphae morphogenesis [31].

Viable cells of *A. actinomycetemcomitans* and *F. nucleatum* from the mono 64.5 h biofilms did not grow out on BHI agar, but they were clearly seen in CLSM images of 64.5 h biofilms. The reason might be that they were in logarithmic decline phase in 64.5 h biofilms, so that they were able to be seen in CLSM images but not detectable on agar plates. Both strains have been related to periodontitis [32, 33] and reported as late colonisers [34], binding to receptors of pioneer colonisers. Our results confirmed the ability of *A. actinomycetemcomitans* and *F. nucleatum* to connect to saliva pellicle without the help of early colonisers, although the adhesion was weak. Karched and coworkers [35] also have proved that the aid of the first colonizers was not a necessary factor for *A. actinomycetemcomitans* and *F. nucleatum* to form biofilms in laboratory conditions.

To date only a limited number of studies have addressed mixed-species biofilms. Results from the current study proved that strains, namely L.GG, *C. albicans, S. sanguinis* and *F. nucleatum*, with poor growth ability in mono-species biofilms demonstrated better abilities of adhesion and reproduction in dual- and/or multi-species biofilms. Similar results have been reported in dual studies, *S. gordonii* and *S. mutans* have shown increased biofilm formation of *C. albicans* [36, 37]. Varposhti et al. [38] have investigated biofilms of six respiratory tract pathogenic bacteria, and their results have indicated that the biofilm formation by two species was significantly greater than its production by any of the single species. Roder and co-workers also found this phenomenon in four species combination isolated from meat chopper and kitchen wall [39]. They suggest that growing with neighbours is, in most cases, advantageous to the productivity of the community [13]. Clinically it seems that biofilms actively attempt to become poly-microbial, apparently to improve their survivability [40]. Behind these phenomena are intra- and inter-species communications, grouping into antagonistic and synergistic effects on microbial community members. The interactions described above clearly show synergistic results for whole communities.

In the last decade, researchers have focused on studying the antagonistic interactions between oral micro-organisms in planktonic and biofilm forms, in vivo and in vitro [41, 42]. To our knowledge, our present study seems to be the first report to explore effects of probiotics on defined oral multi-species biofilms. In our model, L.GG slightly suppressed the increase ratio of *C. albicans* in all groups, markedly restrained the growth of *S. sanguinis* and *F. nucleatum* in 4sp + L.GG group, and slightly reduced the adhesion of *S. mutans* in L.GG + Sm group in in vitro conditions. Our results agree with previous in vitro studies. L.GG was able to inhibit the growth of oral pathogens and opportunistic pathogens in laboratory conditions [19, 20, 43, 44]. This study is an agreement with the previous clinical findings showing that intake of probiotics could significantly reduce the caries risk, gingivitis, periodontal pocket depth and attachment loss, and the counts of yeasts [2, 7–9, 41].

Interestingly, our results showed that *C. albicans* significantly promoted the growth of L.GG. The mechanism is unknown. *C. albicans* was revealed as a basal oral mycobiome member in healthy individuals by multitag 454 pyrosequencing [45], but most studies relate it to diseases, such as oral candidiasis and vaginal yeast infections [46, 47]. Only few studies have reported its contribution to the balance of micro-ecology [11]. One explanation is that lactate, which is generated by L.GG [48], poses harmful effects on itself, but *C. albicans* is able to metabolize it, and to reduce the accumulation and toxic level for L.GG in the environment [37]. Beneficial effects of oral pathogens on lactobacilli have also been reported by Filoche et al. [49] by showing that *Actinomyces* species and *S. mutans* were able to improve the growth of *Lactobacillus*. The mechanism to this is unclear, however.

The pH value of the spent broth varied due to glucose fermentation and other metabolic activities of the micro-organisms on the surface of saliva coated HA discs, in the broth, and on the inner walls of 24-well-plates wells. Our results showed that the number of viable cells in biofilms correlated with lower pH values. *S. mutans, S. sanguinis*, *C. albicans* and L.GG are well-known acid producers [44, 50]. In the adhesion period, inoculated planktonic micro-organisms adjusted themselves to the new conditions in 24-well plates, and attached to the surface of the discs, but the numbers of detected viable cells from the discs were much lower than in the inoculations, thus indicating that the pH value measured at 16.5 h was mainly contributed by planktonic form cells. In the self-development stage, attached biofilm cells were the only micro-organisms in the new wells. After 48 h cultivation biofilms became matured and spent media were filled with planktonic cells. Both biofilms and planktonic cells affected the pH values of the spent media.

## Conclusion

In conclusion, in this in vitro model, L.GG was able to integrate in all oral biofilms on saliva-coated HA discs, and reduced the growth of *S. sanguinis*, *C. albicans*, and lowered the biofilm-forming ability of *F. nucleatum*, but showed only minor effects on the adhesion of *S. mutans. C. albicans* significantly promoted the growth of L.GG. Based on the findings in our study, we could surmise that plausible clinical implication of probiotics could be towards prevention and management of oral infectious diseases by alteration of biofilm composition.

## Methods

### Strains, growth conditions, and inoculum preparation

The commercially available probiotic *Lactobacillus rhamnosus* GG (L.GG) was tested against five oral strains, namely *Streptococcus mutans* (Sm)*, Streptococcus sanguinis* (Ss)*, Aggregatibacter actinomycetemcomitans* (Aa)*, Candida albicans* (Ca)*,* and *Fusobacterium nucleatum* (Fn) (Table 1). *A. actinomycetemcomitans* and *F. nucleatum* were added to increase the complexity for the multi-species groups. All the strains were maintained as frozen stock at −80 °C in 20 % skim milk (Difco™, BD, Becton, Dickinson and Company, Sparks, MD, USA). Before each experiment, strains were cultivated on respective agars (Table 1). Pure colonies of each strain were inoculated in 5 mL corresponding broth, and grown overnight at 37 °C anaerobically.

**Table 1:** Strains and groups involved in this study

a. Strains and growth conditions			
Strain	Origin	Agar/Broth	Growth conditions
*Lactobacillus rhamnosus* GG ATCC 53103 (L.GG)	Valio Ltd., Helsinki, Finland	de Man, Rogosa and Sharpe (MRS)	24 h, 37 °C, 5 % CO_2_
*Candida albicans* ATCC 10231 (Ca)	American Type Culture Collection (ATCC)	Sabouraud	24 h, 37 °C, air
*Streptococcus mutans* ATCC 27351 (Sm)	ATCC	Brain Heart Infusion (BHI)	24 h, 37 °C, 5 % CO_2_
*Streptococcus sanguinis* ATCC 10556 (Ss)	ATCC	BHI	24 h, 37 °C, 5 % CO_2_
*Aggregatibacter actinomycetemcomitans* ATCC 43718 (Aa)	ATCC	BHI	24 h, 37 °C, 5 % CO_2_
*Fusobacterium nucleatum* ATCC 25586 (Fn)	ATCC	Brucella	48 h, 37 °C, in anaerobic condition (mixture of 0.2 % O_2_, 5 % CO_2_, 9.9 % H_2_, 84.9 % N_2_)
b. Group setup of dual- and multi- species biofilm groups and respective agars and cultural conditions to detect viable cells from biofilms			
Groups	Strain(s)	Agars and cultural conditions	
L.GG + Ca	L.GG, Ca	72 h, 37 °C, L.GG: MRS in 5 % CO_2_ condition Ca: Sabouraud in air Sm, Ss, Aa, Fn: BHI in anaerobic condition	
L.GG + Sm	L.GG, Sm		
L.GG + Ss	L.GG, Ss		
4SP	Ss, Aa, Ca, Fn		
4SP + L.GG	Ss, Aa, Ca, Fn, L.GG		
5SP	Ss, Aa, Ca, Fn, Sm		
5SP + L.GG	Ss, Aa, Ca, Fn, Sm, L.GG		

Strains grown overnight in broth were harvested by centrifugation for 10 min at 3,000 × g, room temperature, washed three times with 5 mL 0.9 % NaCl and re-suspended in Biofilm Medium (BM, glucose as carbohydrate source) adapted from Lemos et al. [22]. The suspensions were diluted to an OD_490_ of 0.130 ± 0.010 (similar to McFarland standard No. 1, the concentration of each strain was 10^8^ CFU/mL, but for *C. albicans* it was 10^7^ CFU/mL) by a spectrophotometer (Multiscan Plus, Labsystems, Helsinki, Finland, measured by 200 μL of each well in a 96-well plate). Strains were pooled according to the group setup (Table 1.). Thirteen experimental groups were designed in this study, namely Group L.GG, Group Sm, Group Ss, Group Aa, Group Ca, Group Fn, Group L.GG + Sm, Group L.GG + Ss, Group L.GG + Ca, Group 4sp, Group 5sp, Group L.GG + 4sp, and Group L.GG + 5sp. Physiological saline was used as a negative control. When preparing mixed strains, each strain suspension was pipetted in equal volume in each group.

### Preparation of biofilms

Biofilms were grown on saliva-coated HA discs (Clarkson Chromatography Products, Inc., South Williamsport, PA, USA). The discs were 7.0 mm in diameter and 1.8 mm high. The HA discs were placed in a vertical position by disc holders bent from orthodontic wire according to Lemos et al. [22] with minor changes. The holders and the HA discs were autoclaved after assembling.

To allow formation of a salivary pellicle, each HA disc was placed in a well of a sterile 24-well polystyrene cell culture plate, fully immersed and incubated with 1.8 mL of processed saliva and gently shaken for 4 h at room temperature. The processed saliva was prepared and pasteurized according to Guggenheim et al. [23]. We assessed the efficacy of pasteurization by plating processed saliva samples onto Brucella agar (BBL™, BD, Becton, Dickinson and Company, Sparks, MD, USA, with Vitamin K3 10 ug/mL, Hemin 5 ug/mL, and 5 % defibrinated horse blood from bio TRADING, Mijdrecht, the Netherlands); after 72 h at 37 °C, no CFU were observed on either aerobically or anaerobically incubated plates.

When the saliva pellicle was formed, HA discs were transferred to a new 24-well plate containing 2.5 mL BM and 0.3 mL pooled strains, after two consecutive dip-washes in another 24-well plate filled with 2.8 mL physiological saline per well. The HA discs were then incubated anaerobically at 37 °C for either 16.5 h or 64.5 h. Culture media were renewed at 16.5 h and 40.5 h. The discs were first washed by dipping twice into 2.8 mL physiological saline and then transferred to a new 24-well plate containing 2.8 mL fresh BM per well. Following medium replacement, the plates were returned to the anaerobic incubator.

### Harvesting the biofilms

At the end of their designated incubation times, one portion of the HA discs was taken for counting the cell number on the biofilms. After two dip washes in physiological saline, each HA disc was transferred into a sterile 50 mL polypropylene tube containing 5 mL of physiological saline at room temperature, and vortexed (by Vortex-Genie® 2 mixer, Scientific industries, Inc, Bohemia, N.Y., USA, speed control to position 5) vigorously for 2 min, and sonicated (by Wagner instrusonic, PS-Terä Oy, Lahti, Finland, 90/180 watts) for 5 sec at room temperature [23].

## Examination of harvested cells

Serial dilutions of the sonicated cells were cultivated on agar plates (Table 1). Counts of L.GG, *S. mutans*, *S. sanguinis*, *A. actinomycetemcomitans*, *F. nucleatum*, and *C. albicans* were gained by observation of different colonial morphology on MRS, BHI, and Sabouraud agars (de Man, Rogosa and Sharpe; Brain Heart Infusion; Sabouraud dextrose; Lab M Ltd, Bury, UK) incubated at 37 °C in a 5 % CO_2_ or in anaerobic or air environments. After 72 h, colony forming units (CFU) were counted. Total viable counts in a group were calculated by the sum of the number of each strain.

**Fig. 6:**
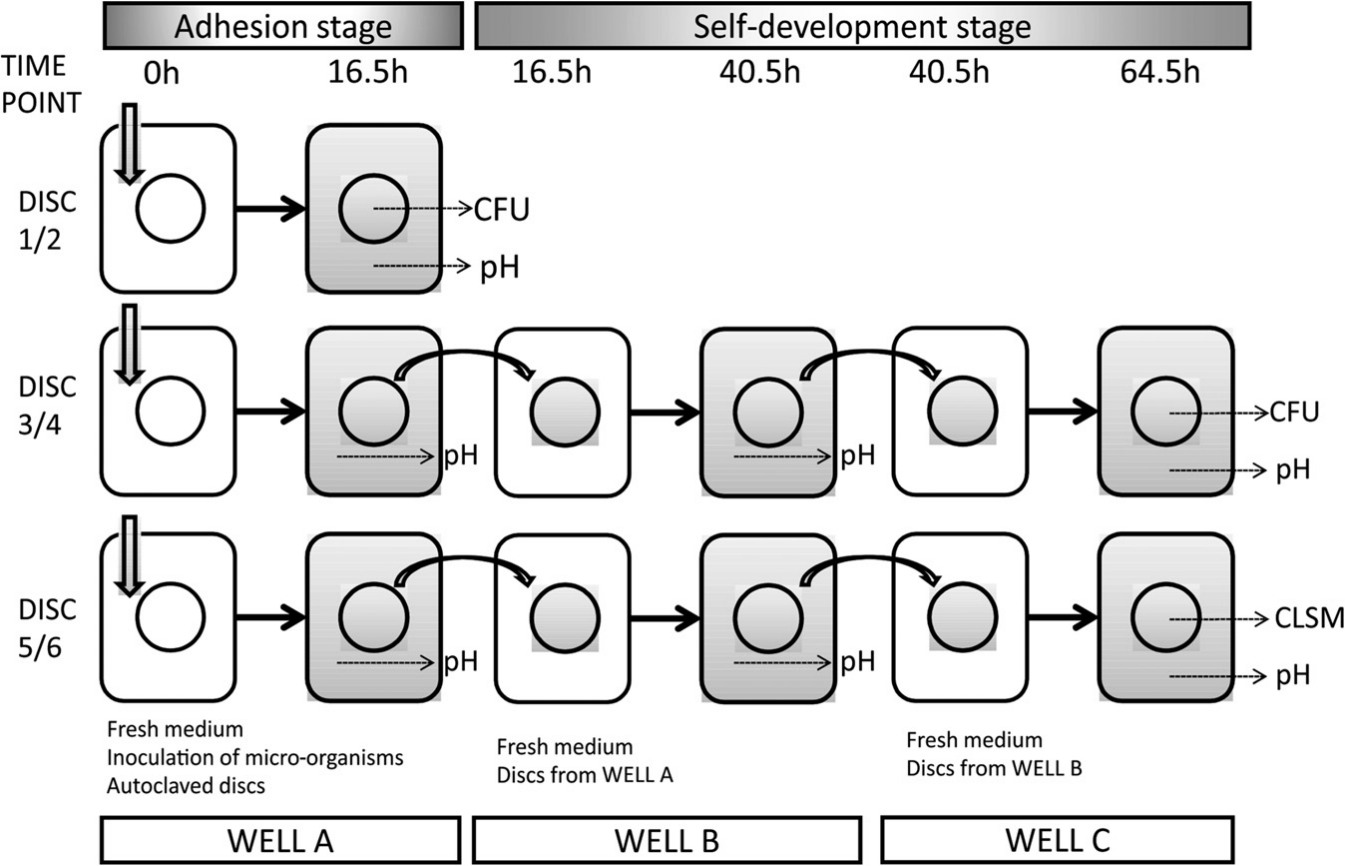
Experimental algorithm for each group. CFU = viable cell counting from HA discs; pH = pH values of spent media; CLSM = observation with confocal laser scanning microscopy

The 64.5 h experiment was separated into two stages for better analysis (Fig. 6). The first 16.5 h was adhesion stage: cell suspensions were inoculated into the wells with saliva-coated HA discs at 0 h (as described in Preparation of biofilms), and planktonic cells started to attach onto the surface of saliva-coated HA discs during this stage. Inoculated volumes of the cell suspensions (IVCS) were recorded and the numbers of viable cells of the 16.5 h biofilms (NVC16.5) were detected. The latter 48 h, from 16.5 h to 64.5 h, was named as self-development stage: biofilms on discs transferred to new wells were the only microbes in the new environment, where biofilm grew and matured. The numbers of viable cells of the 64.5 h biofilms (NVC64.5) were measured. In order to compare abilities of the strains to build the connections to the saliva-coated HA discs in the first stage, adhesion ratio of each strain was calculated in the 13 groups. In self-development stage, the increase ratio, standing for the ability of strains to reproduce themselves, of each strain and total strains were also calculated. The adhesion ratio and increase ratio were calculated by the equations below:

1 $$ adhesion\kern0.5em  ratio=\frac{NVC16.5}{concentration\kern0.5em  of\kern0.5em  cell\kern0.5em  suspension\times IVCS} $$

2 $$ increase\kern0.5em  ratio=\frac{NVC64.5}{average\kern0.5em  of\kern0.5em NVC16.5} $$

NVC16.5/64.5: viable cells of the 16.5/64.5 h biofilms

IVCS: inoculated volume of the cell suspension.

### Confocal Laser Scanning Microscopy (CLSM)

Biofilms on saliva-coated HA discs were stained with LIVE/DEAD *Bac*Light™ Bacterial Viability Kit (catalog number L7007, Molecular Probes™, Life Technologies™, Eugene, Oregon, USA) solution, containing Syto 9 for live cells and propidium iodide for dead cells. Afterwards biofilms were sampled with distilled water. Prepared biofilms were examined with an inverted microscope fitted with an Argon laser (488 nm) for excitation and a TCS SP8 computer-operated confocal laser scanning microscopy system (Leica Microsystems Gmbh Wetzlar, Germany). Filters were set to 493–522 nm for Syto9 and 618–676 nm for propidium iodide. CLSM images were obtained with a × 63 glycerol immersion objective. Each biofilm was scanned at randomly selected areas as a series of vertical optical sections, each section was 0.50 μm thick. Digital images were processed with ImageJ [51].

### pH measurement of spent cultural media

The pH of spent media was measured by pH 1000 L (pHenomenal®, VWR International, Rador, PA, USA) at all three time points when HA discs were transferred into fresh media. The spent media were centrifuged for 10 min, 3,000× *g* prior to pH measurement from the supernatant.

### Statistical analysis

Data are shown as means ± standard deviations. Statistical analyses were performed with IBM SPSS Statistics version 22 for Windows. One way ANOVA and Bonferroni test were used to determine statistical significance. A difference was deemed significant at *P* < 0.05.

## Abbreviations

Aa, *Aggregatibacter actinomycetemcomitans;* ATCC, American Type Culture Collection; BHI, brain heart infusion; BM, biofilm medium; Ca, *Candida albicans;* CFU, colony forming units; CLSM, confocal laser scanning microscopy; Fn, *Fusobacterium nucleatum;* HA, hydroxyapatite; L.GG, *Lactobacillus rhamnosus* GG; MRS, de Man, Rogosa and Sharpe; NS, not significantly; OD_490_, optical density at wave length of 490 nm; Sm, *Streptococcus mutans;* Ss, *Streptococcus sanguinis*

## References

[1] Hill C, Guarner F, Reid G, Gibson GR, Merenstein DJ, Pot B (2014). The International Scientific Association for Probiotics and Prebiotics consensus statement on the scope and appropriate use of the term probiotic. Nat Rev Gastro Hepat.

[2] Karuppaiah RM, Shankar S, Raj SK, Ramesh K, Prakash R, Kruthika M (2013). Evaluation of the efficacy of probiotics in plaque reduction and gingival health maintenance among school children - A Randomized Control Trial. J Int Oral Health.

[3] Nase L, Hatakka K, Savilahti E, Saxelin M, Pönkä A, Poussa T (2001). Effect of long-term consumption of a probiotic bacterium, *Lactobacillus rhamnosus* GG, in milk on dental caries and caries risk in children. Caries Res.

[4] Krasse P, Carlsson B, Dahl C, Paulsson A, Nilsson A, Sinkiewicz G (2006). Decreased gum bleeding and reduced gingivitis by the probiotic *Lactobacillus reuteri*. Swed Dent J.

[5] Slawik S, Staufenbiel I, Schilke R, Nicksch S, Weinspach K, Stiesch M (2011). Probiotics affect the clinical inflammatory parameters of experimental gingivitis in humans. Eur J Clin Nutr.

[6] Twetman S, Derawi B, Keller M, Ekstrand K, Yucel-Lindberg T, Stecksen-Blicks C (2009). Short-term effect of chewing gums containing probiotic *Lactobacillus reuteri* on the levels of inflammatory mediators in gingival crevicular fluid. Acta Odontol Scand.

[7] Teughels W, Durukan A, Ozcelik O, Pauwels M, Quirynen M, Haytac MC (2013). Clinical and microbiological effects of *Lactobacillus reuteri* probiotics in the treatment of chronic periodontitis: a randomized placebo-controlled study. J Clin Periodontol.

[8] Hatakka K, Ahola AJ, Yli-Knuuttila H, Richardson M, Poussa T, Meurman JH (2007). Probiotics reduce the prevalence of oral *Candida* in the elderly - A randomized controlled trial. J Dent Res.

[9] Kraft-Bodi E, Jorgensen MR, Keller MK, Kragelund C, Twetman S (2015). Effect of probiotic bacteria on oral *Candida* in frail elderly. J Dent Res.

[10] Palmer RJ, Gordon SM, Cisar JO, Kolenbrander PE (2003). Coaggregation-mediated interactions of streptococci and actinomyces detected in initial human dental plaque. J Bacteriol.

[11] Wright CJ, Burns LH, Jack AA, Back CR, Dutton LC, Nobbs AH (2013). Microbial interactions in building of communities. Mol Oral Microbiol.

[12] Kolenbrander PE, Palmer RJ, Periasamy S, Jakubovics NS (2010). Oral multispecies biofilm development and the key role of cell-cell distance. Nat Rev Microbiol.

[13] Elias S, Banin E (2012). Multi-species biofilms: living with friendly neighbors. FEMS Microbiol Rev.

[14] Simon-Soro A, Mira A (2015). Solving the etiology of dental caries. Trends Microbiol.

[15] Marsh PD (2003). Are dental diseases examples of ecological catastrophes?. Microbiology.

[16] Söder B, Meurman JH, Söder PÖ. Gingival Inflammation associates with stroke – a role for oral health personnel in prevention: a database study. PLoS ONE. 2015;10(9):e0137142. doi:10.1371/journal.pone.0137142. 10.1371/journal.pone.0137142 PMC4583452 26405803

[17] Anusha RL, Umar D, Basheer B, Baroudi K (2015). The magic of magic bugs in oral cavity: Probiotics. J Adv Pharm Technol Res.

[18] Gruner D, Paris S, Schwendicke F (2016). Probiotics for managing caries and periodontitis: Systematic review and meta-analysis. J Dent.

[19] Teanpaisan R, Piwat S, Dahlen G (2011). Inhibitory effect of oral *Lactobacillus* against oral pathogens. Lett Appl Microbiol.

[20] van Essche M, Loozen G, Godts C, Boon N, Pauwels M, Quirynen M (2013). Bacterial antagonism against periodontopathogens. J Periodontol.

[21] Exterkate RA, Crielaard W, Ten Cate JM (2010). Different response to amine fluoride by *Streptococcus mutans* and polymicrobial biofilms in a novel high-throughput active attachment model. Caries Res.

[22] Lemos JA, Abranches J, Koo H, Marquis RE, Burne RA (2010). Protocols to study the physiology of oral biofilms. Methods Mol Biol.

[23] Guggenheim B, Giertsen E, Schupbach P, Shapiro S (2001). Validation of an in vitro biofilm model of supragingival plaque. J Dent Res.

[24] Filoche SK, Soma KJ, Sissons CH (2007). Caries-related plaque microcosm biofilms developed in microplates. Oral Microbiol Immunol.

[25] Pham LC, Hoogenkamp MA, Exterkate RA, Terefework Z, de Soet JJ, ten Cate JM (2011). Effects of *Lactobacillus rhamnosus* GG on saliva-derived microcosms. Arch Oral Biol.

[26] Pham LC, van Spanning RJM, Roling WFM, Prosperi AC, Terefework Z, Ten Cate JM (2009). Effects of probiotic *Lactobacillus salivarius* W24 on the compositional stability of oral microbial communities. Arch Oral Biol.

[27] Meurman JH, Antila H, Salminen S (1994). Recovery of *Lactobacillus* strain GG (ATCC 53103) from saliva of healthy volunteers after consumption of yogurt prepared with the bacterium. Microb Ecol Health Dis.

[28] Lebeer S, Verhoeven TL, Perea Velez M, Vanderleyden J, De Keersmaecker SC (2007). Impact of environmental and genetic factors on biofilm formation by the probiotic strain *Lactobacillus rhamnosus* GG. Appl Environ Microbiol.

[29] Mashima I, Nakazawa F (2015). Interaction between *Streptococcus* spp. and *Veillonella tobetsuensis* in the early stages of oral biofilm formation. J Bacteriol.

[30] Krzysciak W, Jurczak A, Koscielniak D, Bystrowska B, Skalniak A (2014). The virulence of *Streptococcus mutans* and the ability to form biofilms. Eur J Clin Microbiol Infect Dis.

[31] Theberge S, Semlali A, Alamri A, Leung KP, Rouabhia M (2013). *C. albicans* growth, transition, biofilm formation, and gene expression modulation by antimicrobial decapeptide KSL-W. BMC Microbiol.

[32] Park OJ, Yi H, Jeon JH, Kang SS, Koo KT, Kum KY (2015). Pyrosequencing analysis of subgingival microbiota in distinct periodontal conditions. J Dent Res.

[33] Chahboun H, Arnau MM, Herrera D, Sanz M, Ennibi OK (2015). Bacterial profile of aggressive periodontitis in Morocco: a cross-sectional study. BMC Oral Health.

[34] Sanchez MC, Llama-Palacios A, Blanc V, Leon R, Herrera D, Sanz M (2011). Structure, viability and bacterial kinetics of an in vitro biofilm model using six bacteria from the subgingival microbiota. J Periodontal Res.

[35] Karched M, Bhardwaj RG, Inbamani A, Asikainen S (2015). Quantitation of biofilm and planktonic life forms of coexisting periodontal species. Anaerobe.

[36] Bamford CV, d'Mello A, Nobbs AH, Dutton LC, Vickerman MM, Jenkinson HF (2009). *Streptococcus gordonii* modulates *Candida albicans* biofilm formation through intergeneric communication. Infect Immun.

[37] Metwalli KH, Khan SA, Krom BP, Jabra-Rizk MA. Streptococcus mutans, Candida albicans, and the human mouth: a sticky situation. PLoS Pathog. 2013;9(10):e1003616. doi:10.1371/journal.ppat.1003616. 10.1371/journal.ppat.1003616 PMC3798555 24146611

[38] Varposhti M, Entezari F, Feizabadi MM (2014). Synergistic interactions in mixed-species biofilms of pathogenic bacteria from the respiratory tract. Rev Soc Bras Med Trop.

[39] Roder HL, Raghupathi PK, Herschend J, Brejnrod A, Knochel S, Sorensen SJ (2015). Interspecies interactions result in enhanced biofilm formation by co-cultures of bacteria isolated from a food processing environment. Food Microbiol.

[40] Wolcott R, Costerton JW, Raoult D, Cutler SJ (2013). The polymicrobial nature of biofilm infection. Clin Microbiol Infect.

[41] Glavina D, Gorseta K, Skrinjaric I, Vranic DN, Mehulic K, Kozul K (2012). Effect of LGG yoghurt on *Streptococcus mutans* and *Lactobacillus* spp. salivary counts in children. Coll Antropol.

[42] Samot J, Badet C (2012). Antibacterial activity of probiotic candidates for oral health. Anaerobe.

[43] Lee SH, Kim YJ (2014). A comparative study of the effect of probiotics on cariogenic biofilm model for preventing dental caries. Arch Microbiol.

[44] Jiang Q, Stamatova I, Kari K, Meurman JH (2015). Inhibitory activity in vitro of probiotic lactobacilli against oral *Candida* under different fermentation conditions. Benef Microbes.

[45] Ghannoum MA, Jurevic RJ, Mukherjee PK, Cui F, Sikaroodi M, Naqvi A, et al. Characterization of the oral fungal microbiome (mycobiome) in healthy individuals. PLoS Pathog. 2010;6(1):e1000713. doi:10.1371/journal.ppat.1000713. 10.1371/journal.ppat.1000713 PMC2795202 20072605

[46] Dangi YS, Soni ML, Namdeo KP (2010). Oral Candidiasis: a Review. Int J Pharm Pharm Sci.

[47] Calderone RA, Fonzi WA (2001). Virulence factors of *Candida albicans*. Trends Microbiol.

[48] Manome A, Okada S, Uchimura T, Komagata K (1998). The ratio of L-form to D-form of lactic acid as a criteria for the identification of lactic acid bacteria. J Gen Appl Microbiol.

[49] Filoche SK, Anderson SA, Sissons CH (2004). Biofilm growth of *Lactobacillus* species is promoted by *Actinomyces* species and *Streptococcus mutans*. Oral Microbiol Immunol.

[50] Lahtinen S, Ouwehand AC, Salminen S, von Wright A. Lactic acid bacteria: microbiological and functional aspects. In: 4th edn. Boca Raton: Taylor & Francis Group; 2012.

[51] Schneider CA, Rasband WS, Eliceiri KW (2012). NIH Image to ImageJ: 25 years of image analysis. Nat Methods.

